# Supplementary material for: Going off the rails: Impaired coherence in the speech of patients with semantic control deficits
Source: Neuropsychologia. 2020 Sep;146:107516. doi: 10.1016/j.neuropsychologia.2020.107516 (PMC7534040; doi:10.1016/j.neuropsychologia.2020.107516)
Supplement: Multimedia component 1 [file mmc1.docx]

**Supplementary Materials**

*
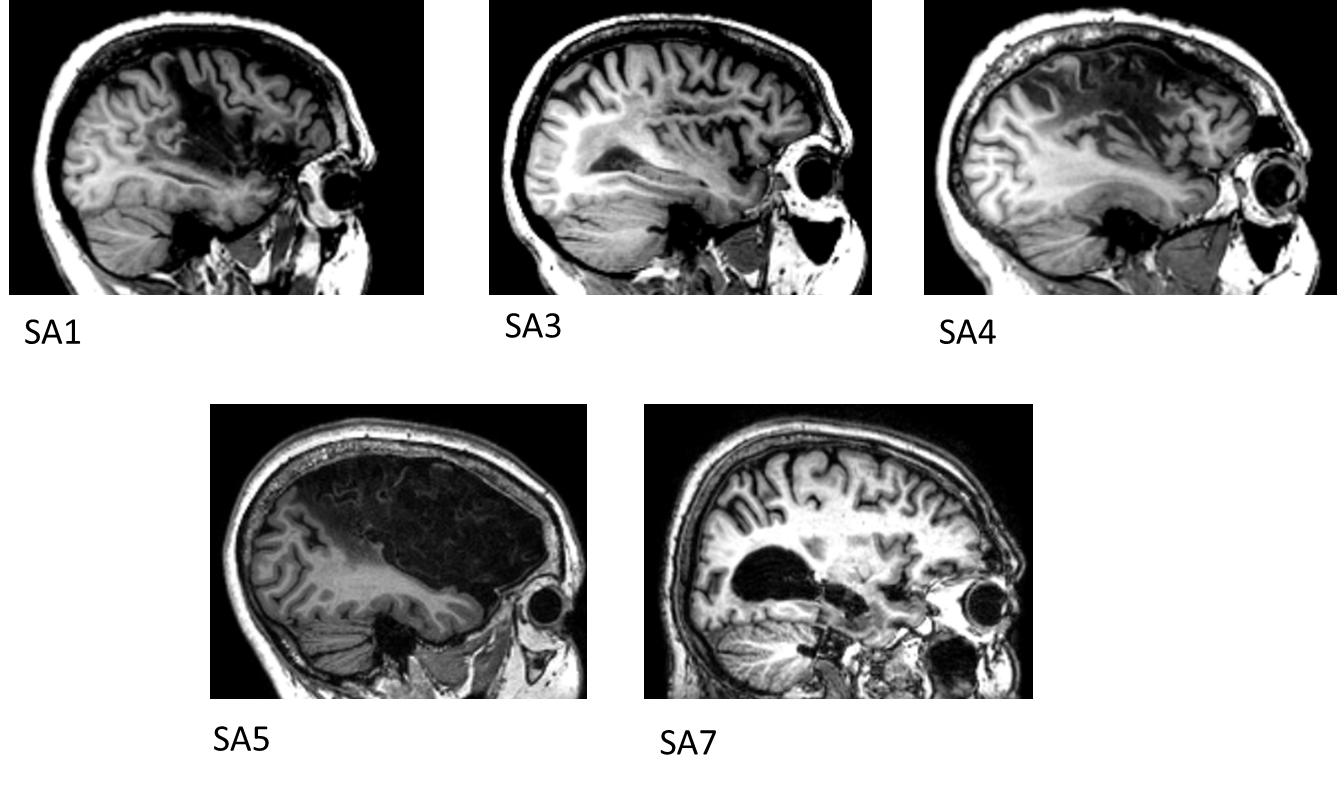
*

*Supplementary Figure 1: Sagittal sections of patients’ MRI scans*

*
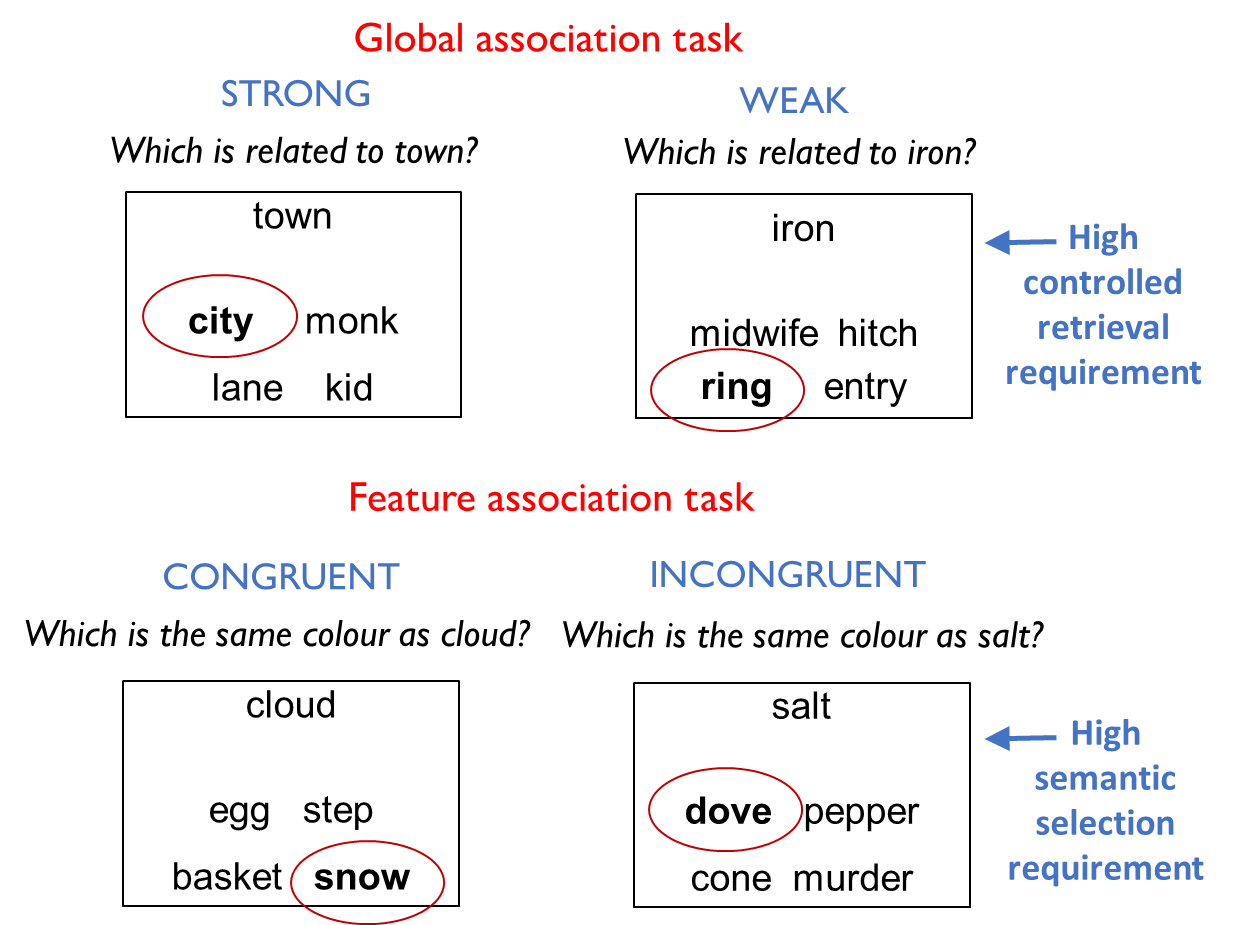
*

*Supplementary Figure 2: Example trials from semantic control tasks*

*The correct response is highlighted in each case.*

*
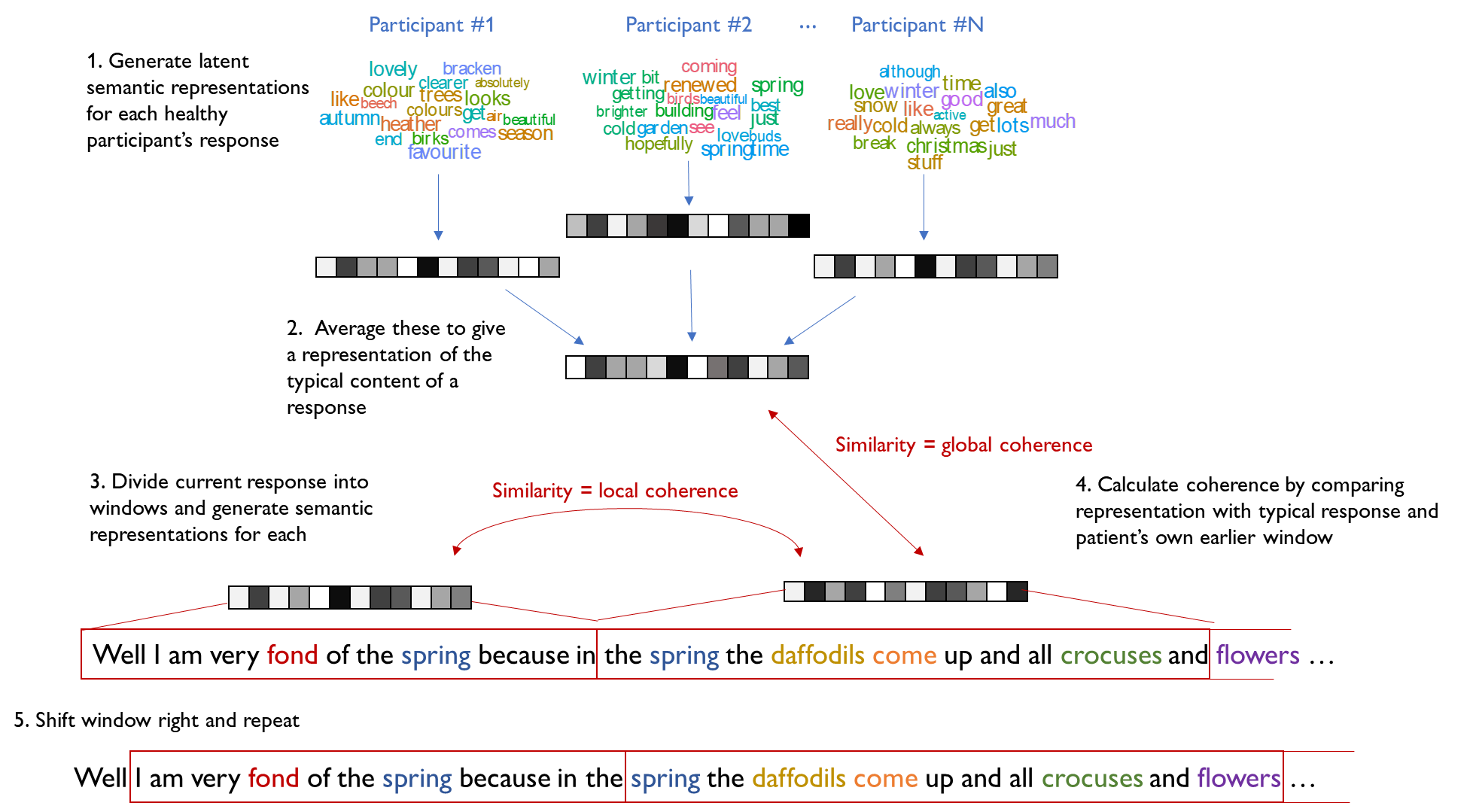
*

*Supplementary Figure 3: Process for computing coherence in speech samples*

Details of additional speech measures

Proportion closed-class words: The proportion of words in each response whose part-of-speech was classified as closed-class. Closed-class words included pronouns, numbers, prepositions, conjunctions, determiners, auxiliaries and some adverbs.

Type: token ratio (TTR): This is the ratio of unique lexical items (types) produced to total words (tokens) spoken. A higher value indicates greater lexical diversity. Because TTR is highly dependent on response length, a moving window approach was adopted to control for the number of words included in the analysis ([Covington and McFall, 2010](#_ENREF_14)). For each response, the first 50 words were first considered and a TTR calculated over these. The window was then moved forward one word and a new TTR calculated, and this process was repeated until the end of the response was reached. A mean TTR was then computed across all windows.

Mean noun frequency: Frequencies in the SUBTLEX-UK database ([Van Heuven *et al.*, 2014](#_ENREF_54)) were obtained for all words tagged as nouns and an average calculated (over tokens) for each response.

Mean noun concreteness: Concreteness ratings for nouns were obtained from Brysbaert et al. ([2014](#_ENREF_9)).

Mean noun age of acquisition (AoA): Estimates of AoA for nouns were obtained from the norms of Kuperman et al. ([2012](#_ENREF_39)).

Mean noun semantic diversity (SemD): SemD values for nouns were obtained from Hoffman et al. ([2013](#_ENREF_26)). SemD is a measure of variability in the contextual usage of words. Words with high SemD values are used in a wide variety of contexts and thus more variable and less well-specified meanings.

Mean noun number of phonemes: The length of all nouns (in phonemes) was also calculated.

Examples of patients’ responses

Patient SA3: Describe the steps you would need to take if going somewhere by train.

Pack a suitcase, get a taxi to the station. And make up, your make up, clothes and sun-tan cream. Get on a train. To London. Went on, went on, went to London Eye in London, went to the barrier reef, no, flood barrier. Went to tea, art exhibition, art exhibition where the gas works were. Went to see Buckingham Palace, went to see man, man, the Mall, went to see [indistinguishable]. Went to see, went in House of Commons, and went to the Westminster Abbey, had to put my shoes on, had to get our shoes, he did. You caught me with my feet. I did have seven, and went in April. And [friend’s name] was there. [Name] with us. Went to see York.

Patient SA7: Why do people go to Scotland on holiday?

Mainly because it’s beautiful countryside. Lovely rivers, beautiful mountains. It just very calming. There’s no stress, no mither. So many places to go around and see. New towns. You can go then, spend a couple of hours having a look round and you see photographs and pictures, going, coming out maybe during the twenties, showing you people and how they lived and what they were dressed in. What were their jobs? And this, these photograph album used to go round and do that when you’re out in the town, any town really. That’s where you’ll find this kind of information, which is really really interesting

Patient SA4: Which is your favourite season and why?

Autumn. Because I like the freshness of it and it, well, it’s been a long hot summer and the freshness of autumn, it. I remember when the kids were little and I used to say you’ve got some roses in your cheeks because we used to go out on long autumn walks and I used to like that. But now they’re grown and I say oh, you haven’t got any roses in your cheeks. Yes. But, and I think it’s fun for the dogs because they don’t like long hot summers and it cools down, you know, in autumn.

Patient SA6: Describe the steps you would need to take if going somewhere by train.

Yes, and the train is busy or empty and, and, and London or Manchester or Scotland, Glasgow. [indistinguishable] Fantastic. And, and four five hours and London or train and speeding, one hour, no, one hour. Oh god, yes, yes, yes. And Edinburgh beautiful but Glasgow, no. Beautiful, Edinburgh, fantastic. But Glasgow, [indistinguishable] and coal if they get it. But air peaty, west and forty miles, beautiful. But raining, raining, raining, raining, raining, the two days and sun, sun shining but sun shines winning, winning, winning similar. But Edinburgh west, east, cold but exciting.

Results using only first 60 seconds of each patient’s response

As noted in the main text, we placed no time limit on patients’ response while controls were subject to a 60s time limit. To confirm that this procedural difference was not responsible for the effects we observed, here we reproduce our main analyses using only the speech produced by patients in the first 60s of each response.


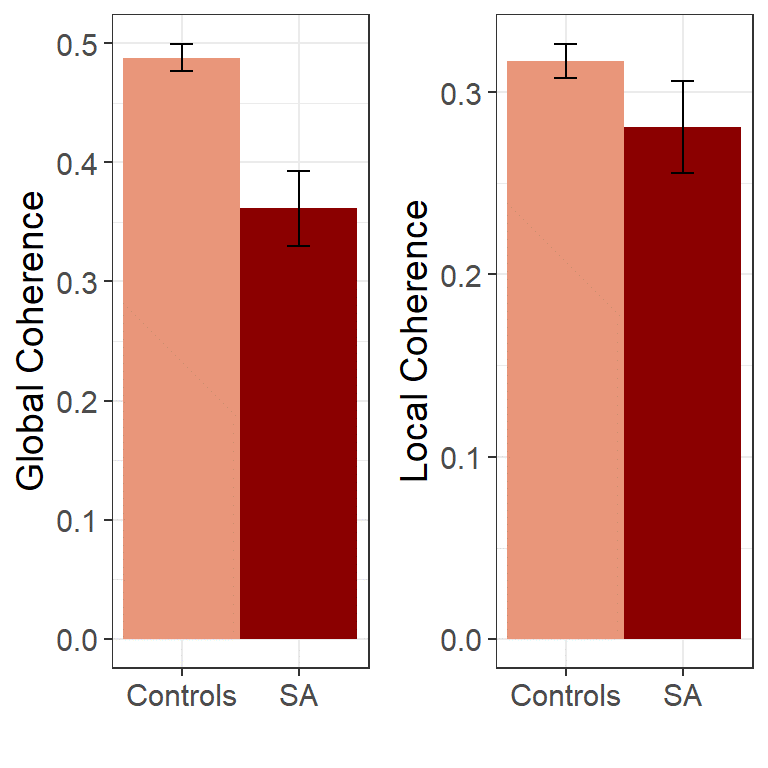


*Supplementary Figure 4: Mean coherence levels for SA patients and controls, based on the first 60s of each response. Bars indicate one standard error of the mean. SA = stroke aphasia. Linear mixed models indicated a significant effect of group on GC (t(16.6) = 4.97, p < 0.001) but no effect on LC (t(19.4) = 1.16, p = 0.26).*


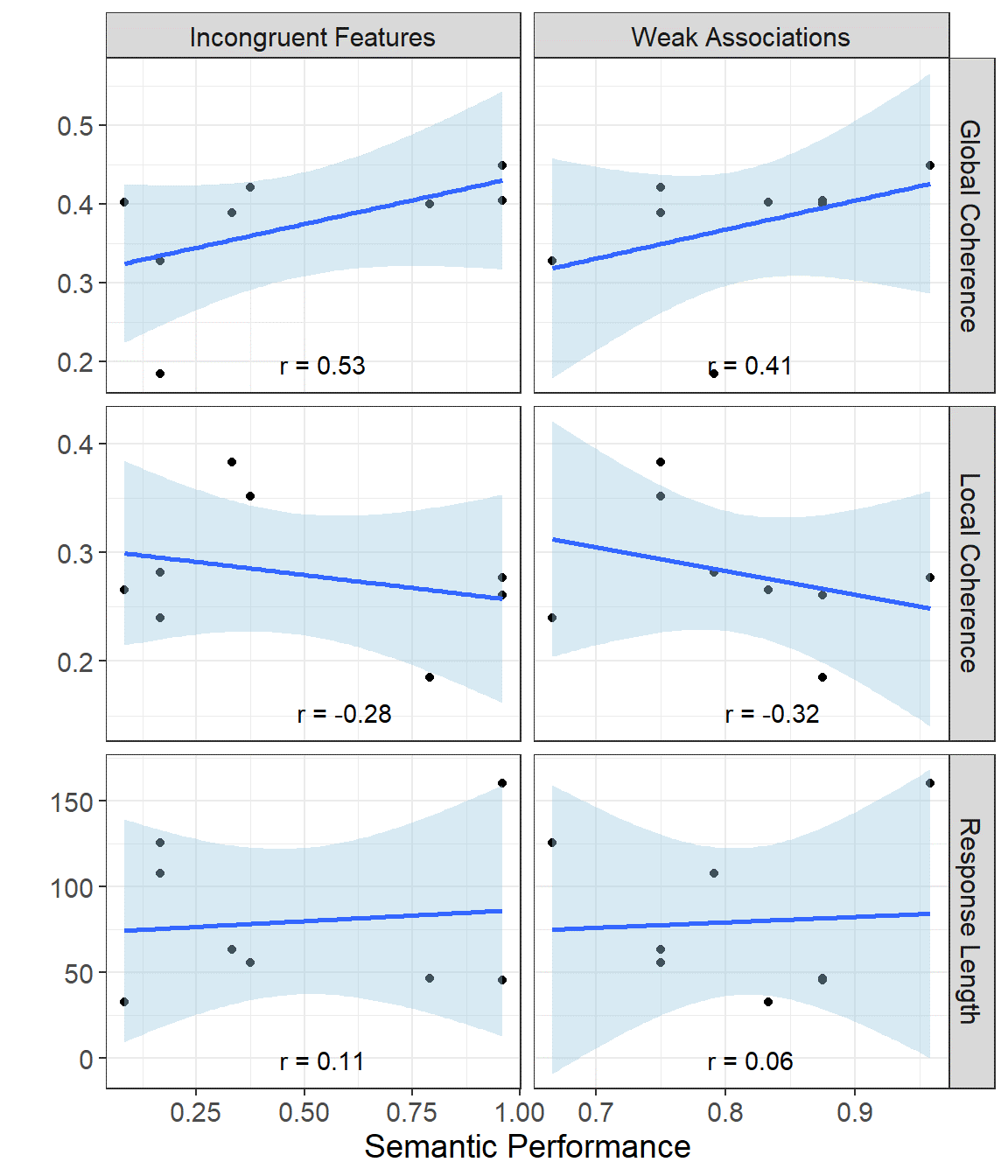


*Supplementary Figure 5: Relationships between coherence, response length and semantic control performance in the patients, based on only first 60s of each response*
